# Discovery of Hub Genes Involved in Seed Development and Lipid Biosynthesis in Sea Buckthorn (*Hippophae rhamnoides* L.) Using UID Transcriptome Sequencing

**DOI:** 10.3390/plants14152436

**Published:** 2025-08-06

**Authors:** Siyang Zhao, Chengjiang Ruan, Alexey A. Dmitriev, Hyun Uk Kim

**Affiliations:** 1 Institute of Plant Resources, Key Laboratory of Biotechnology and Bioresources Utilization, Ministry of Education, Dalian Minzu University, Dalian 116600, China; zsy@dlnu.edu.cn; 2 Engelhardt Institute of Molecular Biology, Russian Academy of Sciences, 119991 Moscow, Russia; alex_245@mail.ru; 3 Department of Bioindustry & Bioresource Engineering, Sejong University, Seoul 05006, Republic of Korea; hukim64@sejong.ac.kr

**Keywords:** *Hippophae rhamnoides* L., seed development, lipid biosynthesis, UID RNA-seq, WGCNA, transcription factors

## Abstract

Sea buckthorn is a vital woody oil species valued for its role in soil conservation and its bioactive seed oil, which is rich in unsaturated fatty acids and other compounds. However, low seed oil content and small seed size are the main bottlenecks restricting the development and utilization of sea buckthorn. In this study, we tested the seed oil content and seed size of 12 sea buckthorn cultivars and identified the key genes and transcription factors involved in seed development and lipid biosynthesis via the integration of UID RNA-seq (Unique Identifiers, UID), WGCNA (weighted gene co-expression network analysis) and qRT-PCR (quantitative real-time PCR) analysis. The results revealed five cultivars (CY02, CY11, CY201309, CY18, CY21) with significantly higher oil contents and five cultivars (CY10, CY201309, CY18, CY21, CY27) with significantly heavier seeds. A total of 10,873 genes were significantly differentially expressed between the S1 and S2 seed developmental stages of the 12 cultivars. WGCNA was used to identify five modules related to seed oil content and seed weight/size, and 417 candidate genes were screened from these modules. Among them, multiple hub genes and transcription factors were identified; for instance, ATP synthase, ATP synthase subunit D and Acyl carrier protein 1 were related to seed development; plastid–lipid-associated protein, acyltransferase-like protein, and glycerol-3-phosphate 2-*O*-acyltransferase 6 were involved in lipid biosynthesis; and transcription factors *DOF1.2*, *BHLH137* and *ERF4* were associated with seed enlargement and development. These findings provide crucial insights into the genetic regulation of seed traits in sea buckthorn, offering targets for future breeding efforts aimed at improving oil yield and quality.

## 1. Introduction

Sea buckthorn (*Hippophae rhamnoides* L.), a perennial shrub or small tree belonging to the Elaeagnaceae family, is mainly distributed in the temperate regions of the Eurasian continent [1]. As an important woody plant for soil and water conservation and desertification control, it can grow on marginal land types such as sandy land, barren mountains and saline–alkali land, meaning that it can avoid land competition with field oil crops [2]. Sea buckthorn seed oil contains more than 140 bioactive components, including unsaturated fatty acids, flavonoids, organic acids, alkaloids, sterols, triterpenes, and various vitamins, which have high medicinal and edible value [3]. Sea buckthorn seed oil constitutes a functionally distinct botanical oil with clinically validated dermatological, cardiometabolic, and anti-inflammatory benefits [4]. Its therapeutic efficacy arises from synergistic interactions between high-value fatty acids (notably, palmitoleic acid) [5], tocopherols/tocotrienols, and flavonoid glycosides, positioning it as a promising nutraceutical and cosmeceutical agent [6]. With the increase in the global consumption of sea buckthorn seed oil, the demand gap is widening. The main reasons for the low yield of seed oil of sea buckthorn are its small seeds and low seed oil content [7]. There are significant differences in seed size, grain weight, and seed oil content among different cultivars of sea buckthorn [8]. The mining and comparative analysis of key genes during the seed development of different cultivars of sea buckthorn may be helpful in understanding the regulatory mechanisms of seed development and lipid biosynthesis, which are important for improving the yield and quality of its seed oils.

Seed size and oil content are crucial indexes in the evaluation of yield and quality in plants. To date, the genes regulating seed size and oil content have been identified in various plants, including *Arabidopsis*, soybean, maize and peanut. *GmST05* [9], *GmAP2-1/4* [10] and *AtSHB1* [11] increased seed size/weight when overexpressed. *OsFBN1* overexpression increased the boosts tiller number but compromised panicle development, grain-filling, and jasmonate signaling under heat stress [12].

The targeted overexpression of transcription factors enhances seed size and weight in soybean and rice. *GmPLATZ* [13] and *bHLH* [14] directly increased seed size/weight when overexpressed or expressed in specific developmental stages. *Dof* transcription factors might modulate grain quality through ethylene and jasmonate signaling [15]. The overexpression of *LuWRI1a* increased seed weight/size and oil content in *Arabidopsis* and increased seed oil reserves in transgenic flax [16]. The transcription factor inhibited the expression of downstream auxin genes, regulating silique cell wall development and seed weight in rapeseed [17].

WGCNA is an efficient method for analyzing and mining high-throughput sequencing data, which can accurately identify hub genes, co-expression modules and regulatory networks that are essential for seed development and lipid metabolism [18]. In the WGCNA, the genes between the samples were divided into modules according to a certain group of co-expressed genes, and then these modules and phenotype data were used to cluster and find the hub genes in the module. In soybean, 946 protein-related and 4,815 fatty acid-associated hub genes were identified via the WGCNA, including known regulators such as *VPS35*, *ABI3b*, and *LEC1d* homologs, as well as novel candidates potentially bridging *WRI1* and *LEAF* networks for oil/protein accumulation [19]. Similarly, in peanut and maize, WGCNA linked seed weight/size traits to modules containing auxin-responsive *ZmARF12* (a cell division repressor) and ribosomal/transcription factor (TF) hubs, demonstrating its power to uncover conserved and species-specific regulatory mechanisms governing seed composition and morphology [20,21].

High-throughput sequencing technology has been applied to identify key genes involved in seed development and lipid metabolism in sea buckthorn [22]. High-throughput RNA sequencing revealed dynamic gene expression patterns during sea buckthorn seed development, identifying 1737 lipid metabolism-related unigenes and key regulators such as delta9-desaturases (*ACP-Δ9D*), *DGAT1*, and *WRI1* that orchestrate fatty acid biosynthesis and triacylglycerol accumulation [8,23]. Integrated small RNA and transcriptome sequencing further uncovered miRNA–transcription factor co-regulatory networks, including 27 miRNAs and 31 transcription factors linked to lipid synthesis and seed development, with critical hubs including miR164d-*ARF2* and novelmiRNA-58-*GPD1* modulating lipid precursors and seed oil content [24]. However, genes related to sea buckthorn seed development and oil accumulation using UID RNA-seq analysis and multiple cultivars have yet to be systematically explored. Very few studies employ WGCNA to investigate genes associated with seed development and oil accumulation in sea buckthorn.

In this study, seeds from 12 sea buckthorn cultivars at two developmental stages were collected; then, UID RNA-seq and WGCNA were performed to identify the key genes and transcription factors regulating seed development and lipid biosynthesis. Furthermore, candidate hub genes were identified through quantitative real-time PCR analysis. The detailed functional investigation of the networks and hub genes and transcription factors could further improve the understanding of the key genes that control seed development and lipid biosynthesis, which are significant for the quality and yield improvement of sea buckthorn.

## 2. Results

### 2.1. Seed Traits

**Figure 1:**
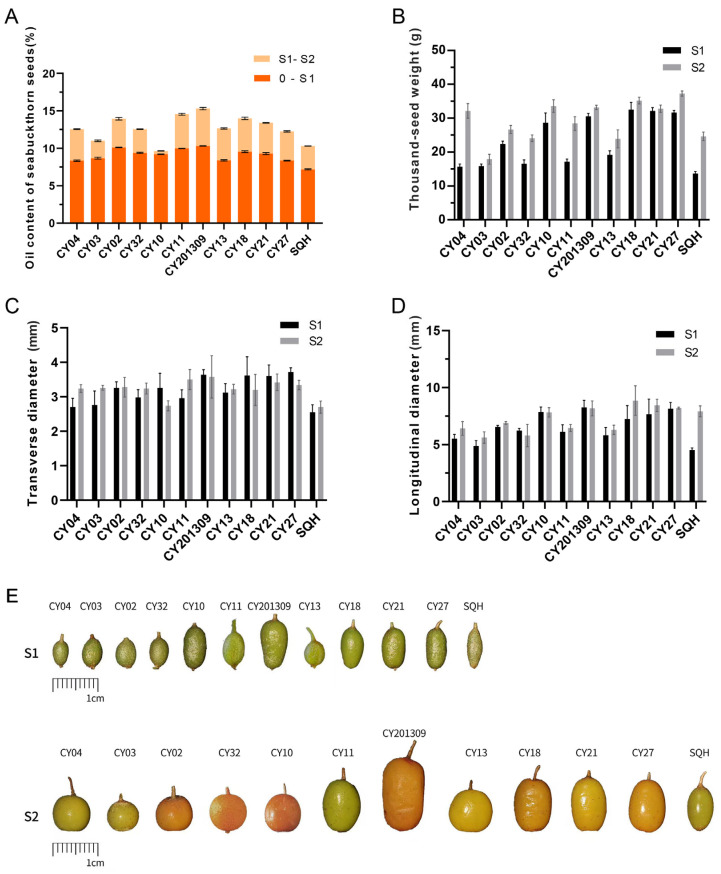
The oil content, thousand-seed weight (TSW) and size of developing sea buckthorn seeds. (**A**) Oil contents in seeds from twelve cultivars, ‘CY04’, ‘CY03’, ‘CY02’, ‘CY32’, ‘CY10’, ‘CY11’, ‘CY201309’, ‘CY13’, ‘CY18’, ‘CY21’, ‘CY27’, and ‘SQH’, at two development stages. (**B**) TSW in seeds from twelve cultivars at two development stages. (**C**) The transverse diameters from twelve cultivars at two development stages. (**D**) The longitudinal diameters from twelve cultivars at two development stages. (**E**) The developmental progress of fruits from 12 cultivars at S1 and S2. Error bars indicate standard deviations of three biological replicates.

At the S1 seed developmental stage, the OC (oil content) of ‘CY02’, ‘CY32’, ‘CY10’, ‘CY11’, ‘CY201309’, ‘CY18’ and ‘CY27’ was higher than that of the other five cultivars (0~S1 vs. S1~S2), while the seed oil contents of ‘CY02’, ‘CY11’, ‘CY201309’, ‘CY18’ and ‘CY21’ were higher than those of the other seven cultivars at the S2 stage (0~S1 vs. S1~S2) (Figure 1A). At the S1 and S2 stages, the oil content of the 12 cultivars ranged from 7.18% ± 0.08 to 10.31% ± 0.04 and 9.65% ± 0.02 to 15.30% ± 0.13, respectively. Compared to the S1 stage, the seed oil contents increased 1.05 to 1.51 fold for all the cultivars at the S2 stage (*p* < 0.01).

At the S1 stage, the TSW (thousand-seed weight) values of ‘CY10’, ‘CY201309’, ‘CY18’, ‘CY21’ and ‘CY27’ were significantly higher than those of the other seven cultivars, while the TSW values of ‘CY04’, ‘CY10’, ‘CY201309’, ‘CY18’, ‘CY21’ and ‘CY27’ were higher than those of the other six cultivars at the S2 stage. From the S1 to S2 stages, the TSWs of each cultivar increased 1.02 to 1.81 fold (Figure 1B). Between stages S1 and S2, OC and TSW were highly significantly positively correlated (*r* = 0.71, *p* < 0.01; *r* = 0.79, *p* < 0.01), while LD (longitudinal diameter) was significantly positively correlated (*r* = 0.65, *p* < 0.05). The TD (transverse diameter) and LD values of the ‘CY10’, ‘CY201309’, ‘CY18’, ‘CY21’ and ‘CY27’ seeds were larger than those of the other seven cultivars (Figure 1C,D). At the S1 stage, TSW showed a highly significant positive correlation with both TD (*r* = 0.96, *p* < 0.01) and LD (*r* = 0.92, *p* < 0.01). Additionally, TSW showed a highly significant positive correlation with LD (*r* = 0.77, *p* < 0.01) but no correlation with TD at the S2 stage. The TD and LD values had a similar expression trend with TSW for developing seeds, which indicated that seed size contributes significantly to weight variation among cultivars. Seeds developed rapidly during the S1 period, while seed oil accumulated quickly during the S2 period. S1 and S2 were the optimal periods for studying seed development and oil synthesis (Figure 1E).

### 2.2. Analysis of UID Transcriptome Data

The final chromosome-scale genome assembly was uploaded to CNCB in the FASTA format with the accession number GWHGEPM00000000.1 and BioProject ID PRJCA042536. In total, about 4.2 billion raw reads were generated, with an average of 59 million reads per sample. After removing adapter sequences, duplicated sequences, ambiguous reads and low-quality reads, a total of 492.01 Gb clean data were generated; 98.34–99.11% of bases had a Q of ≥20, 94.08–96.40% of bases had a Q of ≥30, and the reads had 43.58–46.92% GC content. In our analysis, low-expressed genes were filtered out using a threshold of RPKM < 0.5 (reads per kilobase per million reads) to minimize technical noise, following established RNA-seq analysis standards [25]. This approach is supported by ENCODE guidelines and statistical studies demonstrating improved detection power after filtering [26,27]. There were 10,662 unigenes (98.06%) annotated in the uniprotAc database, 6749 unigenes (62.08%) annotated in the Refseq database, 6759 unigenes (62.16%) annotated in the Pfam database, 9649 unigenes (88.74%) annotated in the interpro database, 1325 unigenes (6.84%) annotated in the eggNOG database, 7992 unigenes (73.50%) annotated in the Gene Ontology (GO) database and 1035 unigenes (9.52%) annotated in the KEGG (Kyoto encyclopedia of genes and genomes) database. The KEGG pathway annotation was used to screen the enriched KEGG data, with *p*-value < 0.05 as the criterion. The KEGG database does not include sea buckthorn, and comparing with closely related species may result in a lower annotation rate.

### 2.3. Identification of Differentially Expressed Genes and Functional Annotation

**Figure 2:**
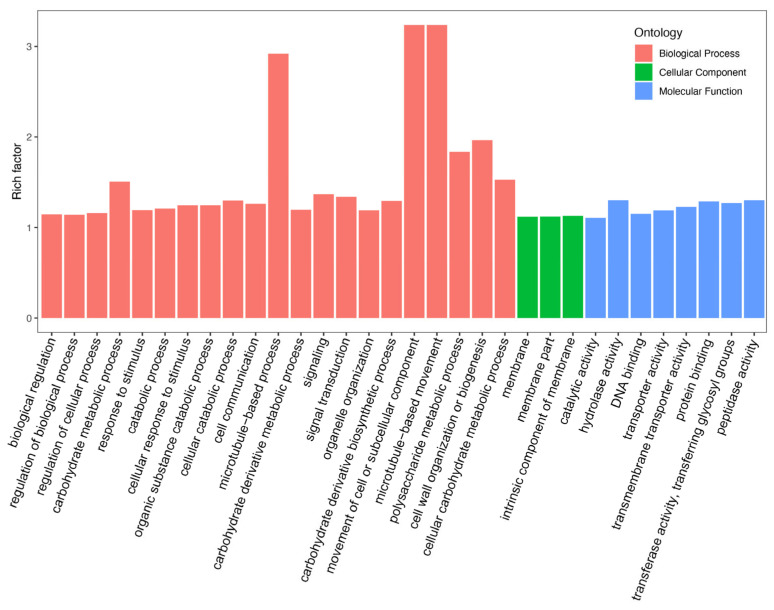
Gene ontology analysis of DEGs.

Differential expression analysis showed that 10,873 DEGs (differentially expressed genes) were screened between the S1 and S2 stages for 12 cultivars, which included 6830 up- and 4043 downregulated genes. The GO terms of DEG-related pathways belong to three categories: biological process (BP), cellular component (CC), and molecular function (MF) (Figure 2). For the BP ontology, the representational enriched terms were photosynthesis, monocarboxylic acid biosynthetic process, and monocarboxylic acid metabolic process. For the MF ontology, the representational enriched terms were cofactor binding, oxidoreductase activity and lyase activity.

**Figure 3:**
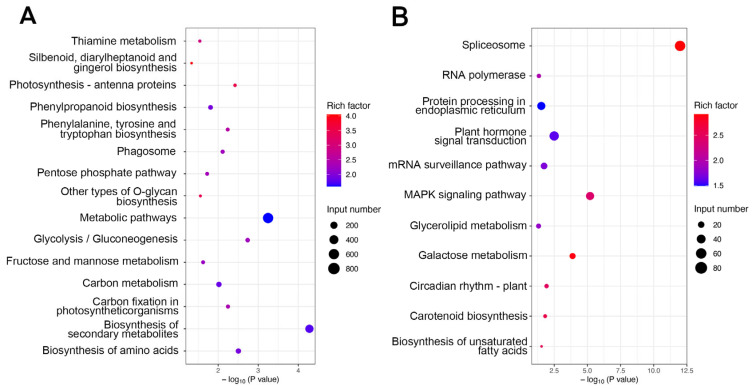
KEGG enrichment analysis of DEGs. Top 20 KEGG up (**A**) and down (**B**) pathways of S1 vs. S2. The abscissa is the P value, and the ordinate is the KEGG path name. The greater the rich factor (*p* < 0.05), the more significant the enrichment level of DEGs in this pathway. The color of the circle represents the degree of enrichment of the rich factor. The size of the circle represents the number of DEGs contained in that KEGG pathway.

DEGs were mapped to the KEGG database, and the enriched KEGG data were filtered (*p* < 0.05). According to the KEGG annotations, 26 KEGG pathways were significantly enriched in upregulated and downregulated genes. The DEGs were mapped to the reference canonical pathways in the KEGG database (Figure 3). As a result, a total of 1990 upregulated DEGs were enriched in 15 pathways. The upregulated DEGs were significantly enriched in the pathways of “Stilbenoid, diarylheptanoid and gingerol biosynthesis”, “Photosynthesis—antenna proteins”, “other types of O-glycan biosynthesis”, “Thiamine metabolism” and “Phenylalanine, tyrosine and tryptophan biosynthesis” (Figure 3A). “Stilbenoid, diarylheptanoid and gingerol biosynthesis” included the key genes *HCT1* (hydroxycinnamoyl transferase 1) and *AK* (adenylate kinase) related to seed development. *HCT* catalyzed the esterification of coumaric acid and quinic acid to produce hydroxycinnamate esters, which were crucial for lignin synthesis. Alterations in HCT activity might impede lignin production, leading to defects in seed cell walls and affecting seed development [28]. *OsAK3* encodes adenylate kinase, which regulates grain size by controlling the growth of lemma cells in spikelets [29]. A total of 354 downregulated DEGs were enriched in 11 pathways. The downregulated DEGs were significantly enriched in the pathways of spliceosome, galactose metabolism, carotenoid biosynthesis, and the biosynthesis of unsaturated fatty acids (Figure 3B).

### 2.4. Differentially Expressed Genes (DEGs) Involved in Seed Development

**Figure 4:**
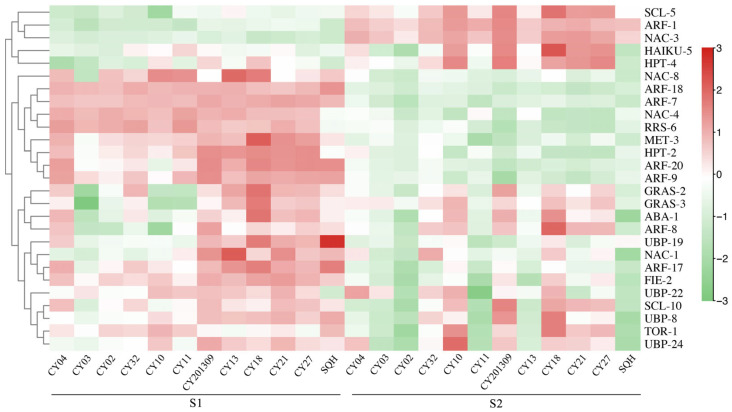
Heatmap analysis of significant DEGs associated with seed development from two developmental stages in twelve cultivars. The expression value (in RPKM) for the DEGs during S1 and S2 development in both cultivars was log2 transformed, and the total RPKM value was greater than 20.

A total of 99 DEGs associated with seed development were identified, including 2 abscisic acid (*ABA*) genes, 2 brassinosteroid insensitive 1 (*BRI1*) genes, 5 choline ethanolamine kinase (*CEK*) genes, 2 cellulose synthase A (*CESA*) genes, 3 cytokinin oxidase (*CKX*) genes, 3 DA1-related protein (*DA1*) genes, 2 fertilization independent endosperm (*FIE*) genes, 3 *GRAS* genes, 4 histone deacetylase (*HDA*) genes, 5 haiku (*IKU*) genes, 4 histidine phosphotransfer (*HPT*) genes, 7 methyltransferase (*MET*) genes, 16 response regulators (*RRS*) genes, 10 scarecrow-like (*SCL*) genes, 1 shatterproof (*SHP*) gene, 2 target of rapamycin (*TOR*) genes, and 28 ubiquitin specific protease (*UBP*) genes, and the total RPKM of each transcript for these genes was greater than 20 (Supplementary Table S1). DEGs were identified using RPKM with the following thresholds: |log_2_FC| ≥ 1 and FDR < 0.05. The functional annotation significantly enriched the pathways of seed development. The heatmap of candidate genes related to seed development showed that most of these genes were highly expressed in both the S1 and S2 stages of 12 cultivars (Figure 4). For five higher TSW cultivars (‘CY10’, ‘CY201309’, ‘CY18’, ‘CY21’ and ‘CY27’), the *RRS-6*, *MET-3* and *HPT-2* genes were highly expressed at the S1 stage, the *SCL-5*, *HPT-4* and *HAIKU-5* genes were highly expressed at the S2 stage, and the *GRAS-2*, *GRAS-3*, *ABA-1*, *UBP-19*, *FIE-2*, *UBP-22*, *SCL-10*, *UBP-8* and *UBP-24* genes were highly expressed at the S1 and S2 stages. The expression levels of the above candidate genes appeared to show a similar trend for TD and LD in most cultivars.

A total of 31 transcription factors associated with seed development were identified, including 20 auxin response factors (*ARFs*), one leafy cotyledon (*LEC*), eight nac transcription factors (*NAC*) and two transparent testa 1 factors (*TT1*). The heatmap of transcription factors related to seed development showed that most of these genes were expressed in both developmental stages S1 and S2 of the 12 cultivars (Figure 4). For five higher TSW cultivars (‘CY10’, ‘CY201309’, ‘CY18’, ‘CY21’ and ‘CY27’), *ARF-7*, *ARF-18*, *NAC-4*, *ARF-9*, *ARF-20* and *LEC* were expressed highly at the S1 stage, *NAC-3* and *ARF-1* were highly expressed at the S2 stage, and *ARF-8*, *NAC-1* and *ARF-17* were highly expressed at the S1 and S2 stages. The expression of the above DEGs appeared to exhibit a similar trend for TD and LD in most cultivars.

### 2.5. Differentially Expressed Genes (DEGs) Involved in Lipid Biosynthesis

**Figure 5:**
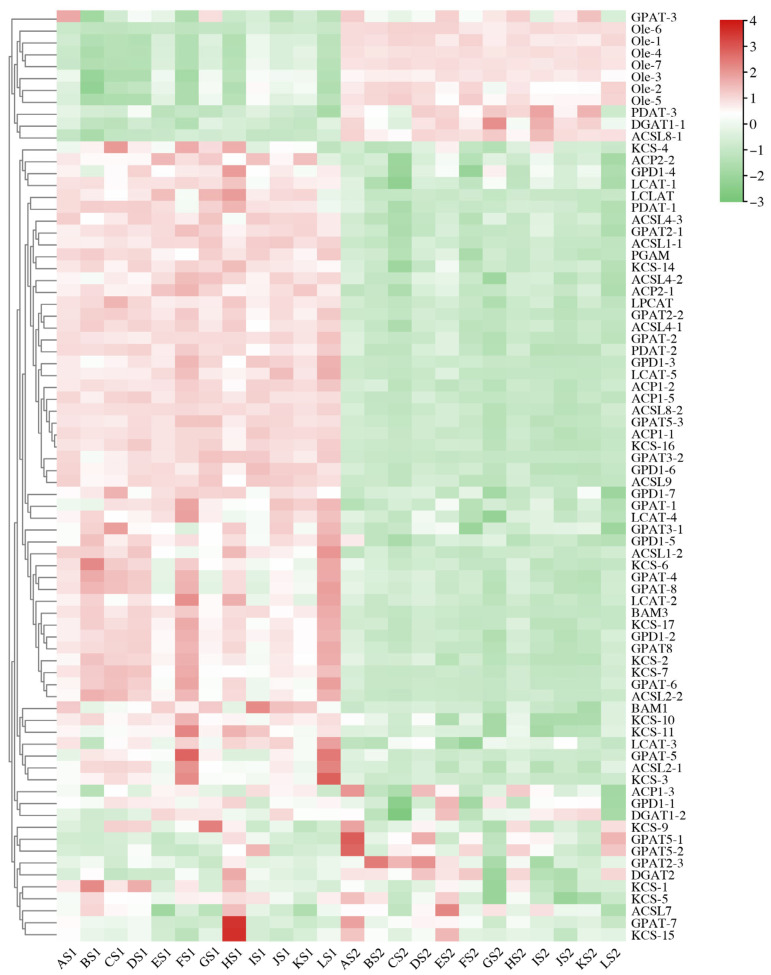
Heatmap analysis of significant DEGs associated with lipid biosynthesis from two developmental stages in twelve cultivars. The expression value (in RPKM) for the DEGs during S1 and S2 development in both cultivars was log2 transformed, and the total RPKM value was greater than 20.

A total of 78 DEGs associated with seed lipid biosynthesis were identified, including 7 glycerol-3-phosphate dehydrogenase (*GPD1*) genes, 17 glycerol-3-phosphate acyltransferase (*GPAT*) genes, 1 lysophosphatidylcholine acyltransferase (*LPCAT*) gene, 1 lysocardiolipin and lysophospholipid acyltransferase (*LCLAT*) gene, 3 diacylglycerol acyltransferase 1 (*DGAT1*) genes, 3 phospholipid diacylglycerol acyltransferase (*PDAT*) genes, 5 lecithin-cholesterol acyltransferase (*LCAT*) genes, 2 threonine-protein kinase BAM (*BAM*) genes, 1 phosphoglycerate mutase (*PGAM*) gene, 7 oleosin (*OLE*) genes, 11 long-chain acyl-CoA synthetase (*ACSL*) genes, 6 acyl carrier protein (*ACP*) *genes* and 14 ketoacyl-CoA synthase (*KCS*) genes (Figure 5). The total RPKM of each transcript for these genes was greater than 20 (Supplementary Table S2). The expression levels of *OLE* family genes exhibited significant differences between the S1 and S2 stages, with a marked surge in expression levels at the S2 stage. The increase was particularly pronounced in some cultivars; for example, for the higher OC cultivars (‘CY11’ and ‘CY201309’), *OLE-6* exhibited significant stage-specific expression differences, particularly in the CY11 and CY201309 (*p* < 0.01). Specifically, its expression level at the S2 stage was 201-fold and 104-fold higher than that at the S1 stage.

The expression levels of *GPD1-6* were the highest among *GPD1* family genes, showing significantly higher values in most cultivars at the S1 stage, compared to S2. *GPD1-2* exhibited significant stage-specific expression differences, particularly in the high-oil-content cultivar CY11, in which its expression level at the S1 stage was 40-fold higher than that at the S2 stage. Both *GPAT-4* and *GPAT-8* demonstrated elevated expression at the S1 stage, with their expression levels in CY11 being 46 and 47 times higher in S1 than in S2, respectively. In contrast, the *DGAT* gene family showed significantly higher expression at S2 compared to S1. *ACSL2-2*, *ACSL4-1*, and *ACSL4-3* displayed relatively high expression levels at the S1 stage and markedly decreased levels at the S2 stage. *KCS-17* exhibited exceptionally high expression at the S1 stage, reaching levels 26-fold greater than those observed at the S2 stage (*p* < 0.05). Conversely, *KCS-10* and *KCS-14* showed increased expression at the S2 stage in some cultivars. Notably, for the CY11 cultivar, the expression levels of *ACP1-1* and *ACP1-2* were dramatically reduced by 20-fold at S2 compared to S1.

**Figure 6:**
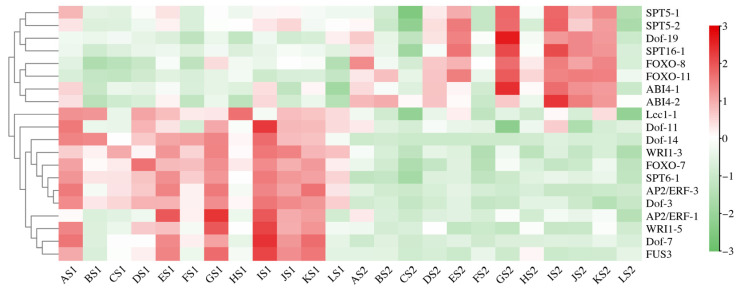
Heatmap analysis of significant TFs associated with lipid biosynthesis from two developmental stages in twelve cultivars. The expression value (in RPKM) for the DEGs during S1 and S2 development in both cultivars was log2 transformed, and the total RPKM value was greater than 20.

A total of 102 differentially expressed TFs were identified between S1 and S2 seed developmental stages of 12 cultivars. The 12 transcription factors of *LEC1-1*, *Dof-11*, *Dof-14*, *WRI1-3*, *FOXO-7*, *SPT6-1*, *AP2/ERF-3*, *Dof-3*, *AP2/ERF-1*, *WRI1-5*, *Dof-7* and *FUS3* were upregulated in high oil content cultivars relative to other cultivars at the S1 stage. A total of eight transcription factors of *SPT5-1*, *SPT5-2*, *SPT16-1*, *Dof-19*, *FOXO-8*, *FOXO-11*, *ABI4-1* and *ABI4-2* were upregulated in high oil content cultivars relative to other cultivars at the S2 stage (Figure 6).

### 2.6. Co-Expression Network Analysis of DEGs by WGCNA

**Figure 7:**
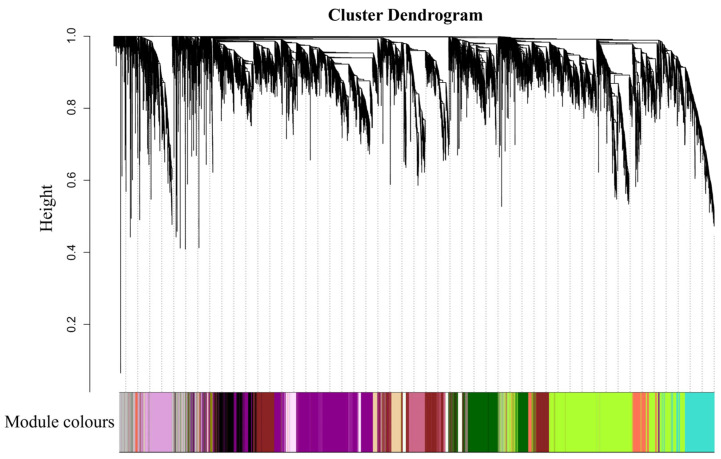
Weighted gene co-expression network analysis and visualization; clustering tree of gene systems based on TOM and the division of modules.

**Figure 8:**
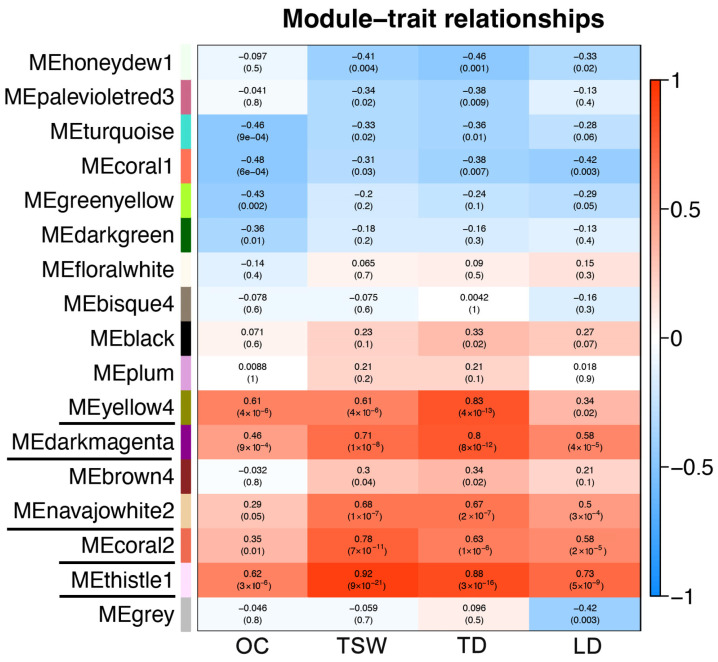
Module–trait correlation heatmap. Each row corresponded to a module, and each column corresponded to a trait. OC: oil content; TSW: thousand-seed weight; TD: transverse diameters; LD: longitudinal diameters.

The WGCNA showed that the gene numbers of different modules varied greatly, ranging from 48 to 2389. A total of 17 different modules were identified (marked with different colors) (Figure 7), 5 of which were significantly associated with OC, TSW, TD and LD. The thistle1 module was significantly positively correlated with TSW (correlation coefficient *r* = 0.92), TD (*r* = 0.88) and LD (*r* = 0.73), the yellow4 module was significantly positively correlated with TD (*r* = 0.83), and the darkmagenta module was significantly positively correlated with TD (*r* = 0.80) (Figure 8).

**Figure 9:**
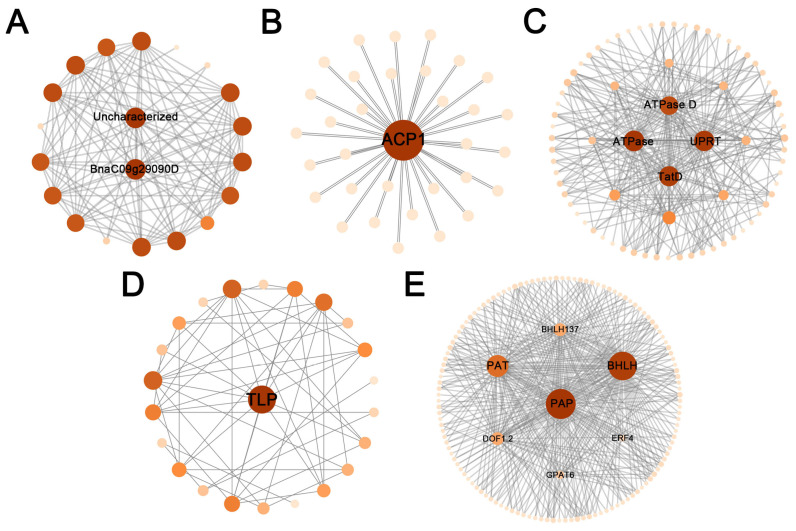
Gene co-expression network analysis in five modules of yellow4 (**A**), darkmagenta (**B**), navajowhite2 (**C**), coral2 (**D**) and thistle1 (**E**).

In the co-expression network of DEGs, 177 hub genes with the highest kME values (kME > 0.95) were screened in five modules (Figure 9). Among these, some hub genes were involved in lipid metabolism, such as *ACP1* (Hic_asm_3.979), *PAP* (Hic_asm_12.1324), *PAT* (Hic_asm_0.74), *GPAT6* (Hic_asm_3.866), and transcription factors *DOF1.2* (Hic_asm_12.3128), *BHLH* (Hic_asm_0.1283), *BHLH137* (Hic_asm_22.1742) and *ERF4* (Hic_asm_3.2216) were associated with seed development.

### 2.7. qRT-PCR of Key DEGs

To validate the precision of the transcriptome sequencing outcomes, 12 hub genes exhibiting differential expression within the trait association module were chosen for qRT-PCR analysis in this investigation. Six hub genes and transcription factors related to seed development were validated: *ACP1* (Hic_asm_3.979), *ATPase* (Hic_asm_12.2935), *ATPase D* (Hic_asm_22.1085), *BHLH* (Hic_asm_0.1283), *DOF1.2* (Hic_asm_12.3128), and *ERF4* (Hic_asm_3.2216) (Figure S1). Six hub genes related to lipid metabolism were validated: *PAP* (Hic_asm_12.1324), *PAT* (Hic_asm_0.74), *GPAT6* (Hic_asm_3.866), *UPRT* (Hic_asm_10.1902), *TatD* (Hic_asm_0.865), and *TLP* (Hic_asm_18.2361) (Figure S2). According to the results of qRT-PCR analysis, the changes in the expression of the selected 12 genes related to lipid biosynthesis and seed development showed a similar tendency, compared to the RNA-seq data, indicating that the UID transcriptomic profiling data were highly reliable (*r* = 0.73, *p* < 0.001).

## 3. Discussion

### 3.1. Key Genes Associated with Seed Development

ATP enzymes play an important role in promoting plant growth and development; they are key membrane proteins responsible for maintaining the proton gradient and ion balance within cells, thus affecting cell expansion and growth [30]. ATPase boosts plant growth by improving photosynthesis via stomatal control [31], enhancing stress resilience (salt/drought) [32], modulating hormone signals [33], and increasing adaptation in transgenic plants [34]. The “MEnavajowhite” module showed strong positive correlations with TSW (*r* = 0.68), TD (*r* = 0.67), and LD (*r* = 0.50). This was due to its enrichment with core genes involved in seed development, such as *ATPase*, *ATPase D*, *UPRT*, and *TatD*. Notably, *ATPase* serves as a hub gene (top 1% connectivity within the module) and its expression level was found to be upregulated by more than three times during the S1 stage, as confirmed by both transcriptome and qRT-PCR analyses. Our finding suggested that the module coordinated the co-expression of seed development genes, with ATP aiding in cell expansion and seed development in sea buckthorn.

ACP plays an important role in seed oil and fatty acid metabolism and affects seed development and maturation by regulating fatty acid synthesis and the desaturation process. The overexpression of the *ACP* gene in *Arabidopsis* significantly alters the fatty acid profile in seeds, increasing the proportion of unsaturated fatty acids, thereby enhancing the quality of seed oils [35]. Studies have shown that *ACP* in *Arabidopsis* can regulate the metabolism of long-chain fatty acids, thereby affecting the plant’s response to low-oxygen conditions and seed development and maturation [36]. The “MEdarkmagenta” module showed strong positive correlations with TSW (*r* = 0.71) and TD (*r* = 0.80). During the S1 stage of sea buckthorn seed development, the expression level of *ACP1* (Hic_asm_3.979) showed a gradual upward trend, decreasing rapidly after the S2 stage. In the S1 stage of seed development, the expression of the *ACP1* gene in CY02, CY201309 and CY18 was rapidly upregulated, which was consistent with the trends of TSW (*r* = 0.85), TD (*r* = 0.79), and LD (*r* = 0.83) increasing gradually at this stage. Our findings suggested that *ACP* contributes to seed development in sea buckthorn.

### 3.2. Key Genes Associated with Lipid Metabolism

The *GPAT* gene family plays an important role in lipid biosynthesis, in which *GPAT6* primarily facilitates triacylglycerol (TAG) synthesis, a process critical for energy storage and membrane integrity [37,38]. The knockout mutant, *gpat6*, caused a massive reduction in seed production [39]. Second, *GPAT6* acts in concert with other acyltransferases to regulate lipid components and affect the fatty acid profile of the plant [40]. The “MEthistle1” module was enriched with core genes of the lipid synthesis pathway, including *GPAT6*, *PAT* and *PAP*, and it exhibits a positive correlation with oil content (*r* = 0.62, *p* < 0.05). *GPAT6* (Hic_asm_3.866) associated with lipid metabolism was identified as both DEGs and hub genes in the module “MEthistle1”; its rapid increase was particularly pronounced in the higher OC cultivars (CY11, CY201309, CY18 and CY21). Furthermore, the expression levels and qRT-PCR results show a consistent trend with changes in oil content. These suggested that *GPAT6* promotes lipid synthesis and accumulation during sea buckthorn seed development.

The *PDAT* gene is involved in TAG synthesis and shows different substrate specificities in different plants, which further affects the quality and composition of oil [41]. In *Brassica napus* [42] and *Gossypium* [43], it was found that the content of oils and the proportion of specific fatty acids were significantly increased by overexpressing *PDAT1* genes from other plants. Through functional studies of different acyltransferases, scientists found that their interaction and substrate selectivity in vegetable oil synthesis have a significant impact on the final oil composition [44]. The pivotal role of acyltransferase-like proteins (PATs) in regulating seed triacylglycerol (TAG) accumulation and seed development has been thoroughly established. Seed-specific overexpression of *DGAT* in wild-type *Arabidopsis* significantly enhanced oil deposition and increased average seed weight, with both traits exhibiting a positive correlation with *DGAT* transcript levels [45]. During the S1 stage of sea buckthorn seed development, expression levels of *PAT* (Hic_asm_0.74) showed gradual upward trends, decreasing rapidly after the S2 stage. In the S1 stage of seed development, the expression of the *PAT* gene in CY10, CY201309, CY18 and CY21 was rapidly upregulated, which is consistent with the trends of TSW, TD, and LD increasing gradually at this stage. This suggests that the *PAT* gene contributes to lipid biosynthesis in sea buckthorn seeds.

### 3.3. Transcription Factors Associated with Seed Development

Transcription factors (TFs) play pivotal roles in orchestrating seed development and grain weight determination. Among these, members of the BHLH family emerge as central regulators, governing seed size, shape, and developmental timing through stage-specific gene expression modulation and protein complex formation [46,47,48,49]. The “MEthistle1” module was enriched with core transcription factors of seed development, including *DOF1.2*, *BHLH* and *ERF4*, and it exhibits a positive correlation with TSW (*r* = 0.92), TD (*r* = 0.88) and LD (*r* = 0.73). In our study, *BHLH* and *BHLH137* showed significantly higher expression in CY201309. *BHLH137* was upregulated in high-TSW cultivars (CY201309 and CY27) relative to other cultivars at the S1 stage. Notably, *BHLH137* exemplified this functional significance in this study. Previous reports have found that its heterologous expression in *Pyrus bretschneideri* promoted cell expansion [50], while interactions with partners such as *PbGIF1* amplified its transcriptional activation capacity. Furthermore, *BHLHs* integrated hormonal signaling, particularly auxin and gibberellin pathways, to fine-tune seed development [51,52]. These multifaceted roles make BHLH proteins a major target for the genetic improvement of seed traits.

*ERF* family transcription factors regulate seed size by interacting with stress-response networks and growth signals [53]. *ERF4* embodies these two functions: it regulates fruit ripening by inhibiting ethylene biosynthesis genes (*ACS1*/*ACO1*) through JAZ-MYC2 interaction [54], while its overexpression induces cell expansion, a mechanism directly implicated in its role in seed development [55]. Thus, ERF proteins link environmental adaptation and developmental programs and potentially enhance seed weight under complex conditions. In summary, *ERF4* may play multiple roles in regulating seed size and weight, involving transcriptional regulation and interactions with other growth factors.

The Dof zinc finger protein family plays an important role in plant growth and development [56,57]. They exhibit tissue-specific expression patterns linked to growth and stress resilience [58,59]. Studies on rice, soybean, and wheat have revealed their effects on root structure and abiotic stress tolerance [60,61], while transgene overexpression enhanced biomass and environmental adaptability [62,63,64]. The elevated expression of *DOF1.2* in specific genotypes such as CY02 and CY201309 in this study highlights its potential role in pathways related to seed size and oil synthesis. The Dof protein might be the hub for integrating cell expansion and oil accumulation. The results provide a new strategy for improving the seed size and oil content of sea buckthorn.

## 4. Materials and Methods

### 4.1. Plant Materials

Twelve sea buckthorn cultivars (*Hippophae rhamnoides* L.) with different phenotypes in seed weight/size and oil content were selected in 2022 in an experimental field of the Dryland Agriculture and Forestry Research Institute (N 41°29′, E 120°22′) in the Liaoning province of China, including ‘CY04’, ‘CY03’, ‘CY02’, ‘CY32’, ‘CY10’, ‘CY11’, ‘CY201309’, ‘CY13’, ‘CY18’, ‘CY21’, ‘CY27’, and ‘SQH’. In total, the 11 elite lines of ‘CY04’, ‘CY03’, ‘CY02’, ‘CY32’, ‘CY10’, ‘CY11’, ‘CY201309’, ‘CY13’, ‘CY18’, ‘CY21’ and ‘CY27’ were selected from wild sea buckthorn by our group, as well as a commercial variety of ‘SQH’ introduced from Russia. From each plant, four primary fruiting branches (two from the sun-exposed canopy and two from shaded sectors) were tagged. All fruits within 20 cm of branch terminals were harvested using sterilized scissors, avoiding mechanical damage. We collected the fruits of sea buckthorn at 30 and 60 days after flowering, considered as the S1 and S2 development stages. Harvested fruits were individually wrapped in pre-sterilized aluminum foil, foil-wrapped samples were submerged in liquid nitrogen for 60 s to ensure the ultrarapid vitrification of tissues. Frozen samples were stored in a −80 °C freezer until analysis.

### 4.2. Determination of Seed Traits

The chloroform–methanol method was used to determine the oil content of sea buckthorn seed at two stages [65]. Then, 5 g of freeze-dried seed powder (M1) was extracted with methanol and chloroform (*v*/*v* 1:2), followed by vortexing or ultrasonication for 20 min. After three repeated extractions, 1/5 volume of KCl solution (*m*/*m*: 0.9%) was added to the pooled extract. The mixture was vortexed for three minutes, and then the solution was allowed to delaminate for five minutes. The bottom layer of the solution was collected into new glass sample bottles (M2) and evaporated to a constant weight (M3) with a rotary evaporator (DLAB, RE100-Pro, Beijing, China). Seeds were dried at 60 ± 2 °C in a desiccator (Carbolite Gero, AX30, Neuhausen, Germany) for four hours until a constant weight was achieved, defined as a mass change ≤ 0.1% between two consecutive weightings after one-hour drying intervals [66]. OC was calculated using the following formula: oil content (%) = (M3 − M2)/M1 × 100%. Three biological replicates were performed for each sample to determine oil content.

TD, LD and TSW were measured as key indicators to evaluate sea buckthorn seed development. A total of 1000 intact seeds were randomly selected to measure TSW, with visual inspections used to exclude damaged or underdeveloped individuals [67]. To ensure operational accuracy, seeds were divided into 10 subgroups of 100 seeds each. Each subgroup was weighed individually, and the total weight was calculated as the sum of all subgroups. Seed weight was determined using an analytical balance (Sartorius, CPA225D, Göttingen, Germany, accuracy ±0.0001 g), calibrated daily with standard weights. TSW was calculated using the formula: TSW (g) = (Total weight of subgroups/Number of subgroups) × 10. Data were expressed as the mean ± standard deviation (SD) from three independent replicate subgroups. The coefficient of variation (CV) was calculated to assess seed weight heterogeneity.

LD and TD were measured using a digital caliper (Mitutoyo, 500-196-30, Utsunomiya-shi, Japan, accuracy ±0.01 mm). LD was defined as the maximum longitudinal axis and TD as the maximum transverse axis perpendicular to length. Each seed was measured three times by the same operator, and the mean value was calculated to minimize operational errors. The coefficient of variation (CV) values for LD and TD were 0.01–0.17 and 0.02–0.17, respectively.

### 4.3. Total RNA Extraction, Library Preparation and UID Transcriptome Sequencing

Total RNA was extracted from developing seeds harvested at the S1 and S2 stages using the Spin Column Plant Total RNA Purification Kit (Sangon Biotech, Shanghai, China). The extracted RNA was subsequently purified using the Dynabeads TM Oligo (dT) 25 mRNA kit (Thermo Fisher Scientific Baltics, UAB, Waltham, MA, USA). The integrity of the RNA was verified through 1.5% agarose gel electrophoresis. Quantification of the qualified RNA samples was conducted using the Qubit 3.0 fluorometer with the Quant-iT RNA Assay Kit, Broad Range (Thermo Fisher, Q10213, Waltham, MA, USA). For the preparation of stranded RNA sequencing libraries, 2 μg of total RNA was utilized, employing the KC-Digital Stranded mRNA Library Prep Kit for Illumina^®^ (Catalog No. DR08502, Wuhan Seqhealth Co., Wuhan, China). The kit mitigates duplication bias during PCR and sequencing processes by employing unique identifiers consisting of eight random bases to label pre-amplified cDNA molecules. Library products ranging from 200 to 500 base pairs were subsequently enriched, quantified, and sequenced using the DNBSEQ-T7 sequencer (MGI Tech Co., Ltd., Shenzhen, China) with the PE150 model.

The initial raw sequencing data underwent quality control using Trimmomatic (version 0.36), whereby low-quality reads were removed and adaptor-contaminated reads were trimmed. Subsequently, the resulting clean reads were processed with proprietary scripts to mitigate the duplication bias introduced during library preparation and sequencing. Specifically, the clean reads were initially clustered based on UID sequences, grouping reads with identical UID sequences into the same cluster. Within each cluster, reads were subjected to pairwise alignment, and those exhibiting sequence identity exceeding 95% were extracted into a new sub-cluster. Following the generation of all sub-clusters, multiple sequence alignment was conducted to derive a consensus sequence for each sub-cluster. Subsequently, any errors and biases introduced by PCR amplification or sequencing were mitigated. Functional annotation of unigene sequences was performed through comprehensive BLASTX (version 2.6.0+) alignment analyses against four major biological databases: the NCBI non-redundant protein database (Nr, release 2023-08) using an E-value threshold of 1 × 10^−5^, the manually curated Swiss-Prot protein database (release 2023_07), the Kyoto Encyclopedia of Genes and Genomes pathway database (KEGG, version 102.0), and the evolutionary classification system of the Cluster of Orthologous Groups database (COG, updated 2023).

### 4.4. RNA-Seq Data Analysis

Transcript or gene expression levels were quantified using RPKM values, which normalize the counts of short sequences by accounting for read depth and transcript length. A pairwise transcriptome comparison was then performed between the control (S1) and experimental groups (S2). DEGs were analyzed utilizing the Edge software version 1.0. Genes exhibiting a false discovery rate (FDR) of less than 0.01 and a fold change of 2.0 or greater were deemed significantly differentially expressed between the control and experimental groups.

### 4.5. KEGG Pathway Enrichment Analysis

The identified DEGs were subsequently annotated using the KEGG database [68], a well-known public resource for metabolic pathways and functional information on gene products (http://www.genome.jp/kegg, accessed on 31 December 2022). Pathway analysis was performed by comparing pathways significantly associated with DEGs to the genomic background. This comparison was achieved by calculating a *p*-value via the hypergeometric distribution, with adjustments for multiple testing accomplished through FDR correction. Concurrently, MapMan software version 3.5.1 was employed to enhance the intuitive visualization of DEGs within the metabolic pathway.

### 4.6. Weighted Gene Co-Expression Network Analysis (WGCNA)

Utilizing input files containing RPKM values for all genes and phenotypic data from 24 samples, the WGCNA package in R software (Version 3.4.4) was applied to identify modules of highly correlated genes [69]. The resulting data were visualized using Cytoscape software (Version 3.6.1). The Pick Soft Threshold function was utilized to determine the optimal soft power, culminating in the construction of the co-expression network at a soft power of *β* = 10 (*R*^2^ = 0.83). We designated a merge CutHeight parameter of 0.25 to consolidate distinct modules exhibiting a similarity exceeding 75%. Combining the transcriptomic and phenotypic data of 24 samples, the WGCNA of the modules was performed using R software. A cluster tree was plotted against the expression of genes, with high correlation among genes within the same cluster. Gene division modules were performed using a dynamic programming algorithm. Subsequently, genes demonstrating analogous expression patterns were amalgamated into the same module. We computed the kME (module eigengene) value to assess the effective connectivity among hub genes. In this investigation, modules with a kME value greater than 0.7 were selected, as these genes more accurately represent the overall expression trend of the module. Ultimately, hub genes and transcription factors within the module were identified based on the kME value and the degree of connectivity of the genes within the network.

### 4.7. Quantitative Real-Time PCR (qRT-PCR) Analysis

Total RNA was extracted from frozen developing seeds at S1 and S2 from 12 cultivars. Complementary DNA (cDNA) synthesis was conducted using 1 μg of total RNA with the HiScript II QRT SuperMix Kit (Vazyme, Nanjing, China), following the manufacturer’s protocol. qRT-PCR was performed in triplicate for three biological replicates. The expression levels of the target genes were normalized using the sea buckthorn genes *UBQ5* as a reference gene. Relative expression levels were determined using the 2^−ΔΔCt^ comparative threshold cycle (Ct) method. All gene-specific primers utilized in this study are listed in Supplementary Table S3.

## 5. Conclusions

Sea buckthorn is a woody oil tree species that is widely distributed in China, India, Russia, and other countries. To solve the challenges of low seed oil content and small seed size in the sea buckthorn industry, this study identified multiple key genes related to the lipid synthesis of sea buckthorn, such as *PAP*, *PAT*, and *GPAT6*. The hub genes *ATPase*, *ATPase D*, and *ACP1* were related to seed development, and transcription factors *DOF1.2*, *BHLH* and *ERF4* were associated with seed growth and development. These findings provide gene resources for sea buckthorn breeding, especially for optimizing oil yields by enhancing seed oil content and seed size.

## Data Availability

The original contributions presented in this study are included in the article/Supplementary Material. Further inquiries can be directed to the corresponding author.

## Supplementary Materials

The following supporting information can be downloaded at https://www.mdpi.com/article/10.3390/plants14152436/s1, Figure S1: The quantitative real-time polymerase chain reaction verification of six hub genes and transcription factors related to lipid biosynthesis. Figure S2: The quantitative real-time polymerase chain reaction verification of six hub genes related to lipid biosynthesis. Table S1: Gene annotation of seed development candidate genes and transcription factors. Table S2: Gene annotation of lipid biosynthesis candidate genes and transcription factors. Table S3: Information of all primer sequences in qRT-PCR.
