# Supplementary material for: Discovery of Hub Genes Involved in Seed Development and Lipid Biosynthesis in Sea Buckthorn (Hippophae rhamnoides L.) Using UID Transcriptome Sequencing
Source: Plants (Basel). 2025 Aug 6;14(15):2436. doi: 10.3390/plants14152436 (PMC12349325; doi:10.3390/plants14152436)
Supplement: Supplementary file 1 [file plants-14-02436-s001.zip › Figure S1.pdf]

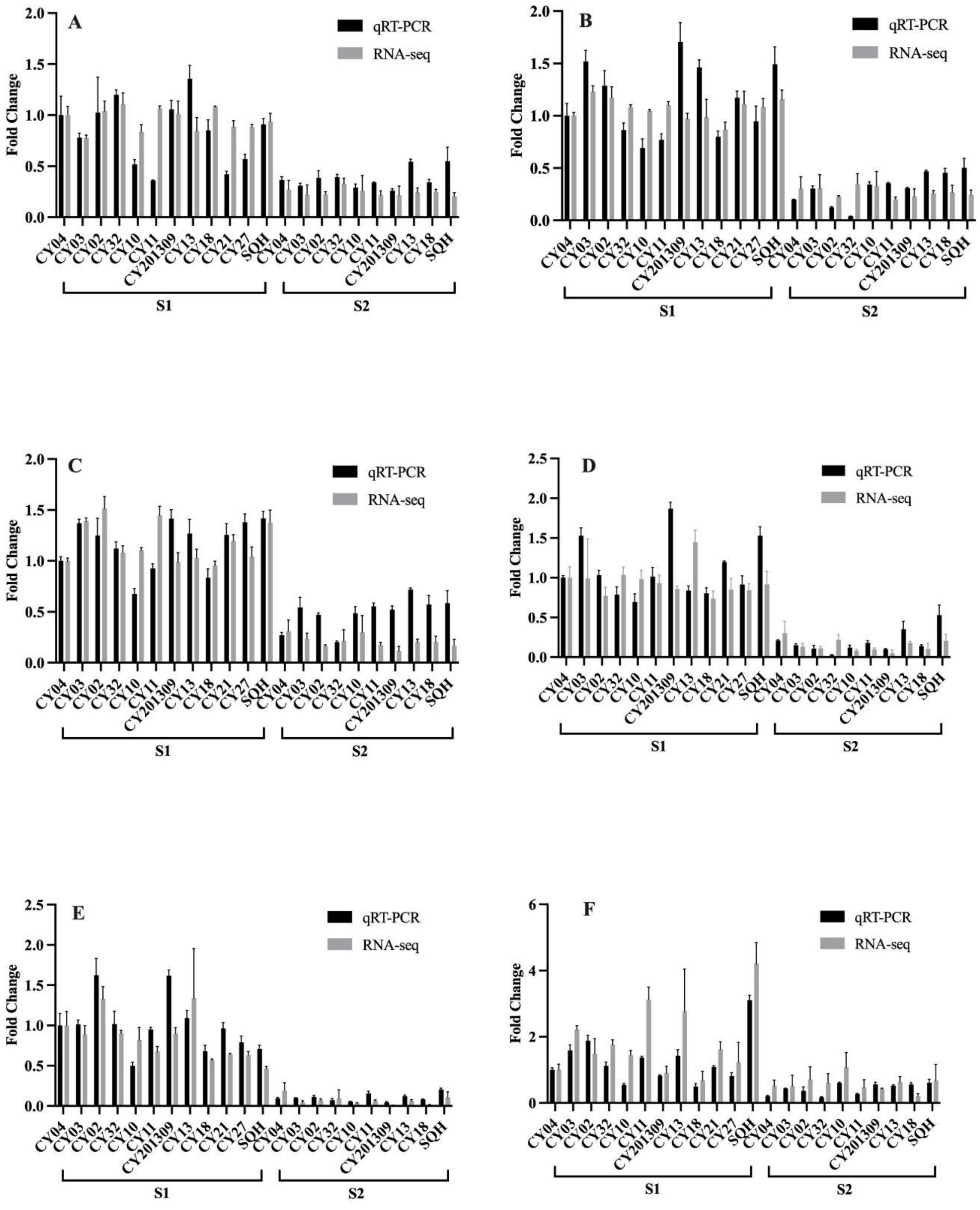

Figure S1. The quantitative real time polymerase chain reaction verification of six hub genes and transcription factors related to lipid biosynthesis. (A) ACP1 (Hic_asm_3.979). (B) ATPase (Hic_asm_12.2935). (C) ATPase D (Hic_asm_22.1085). (D) BHLH (Hic_asm_0.1283). (E) DOF1.2 (Hic_asm_12.3128). (F) ERF4 (Hic_asm_3.2216).
