# Supplementary material for: Discovery of Hub Genes Involved in Seed Development and Lipid Biosynthesis in Sea Buckthorn (Hippophae rhamnoides L.) Using UID Transcriptome Sequencing
Source: Plants (Basel). 2025 Aug 6;14(15):2436. doi: 10.3390/plants14152436 (PMC12349325; doi:10.3390/plants14152436)
Supplement: Supplementary file 1 [file plants-14-02436-s001.zip › Figure S2.pdf]

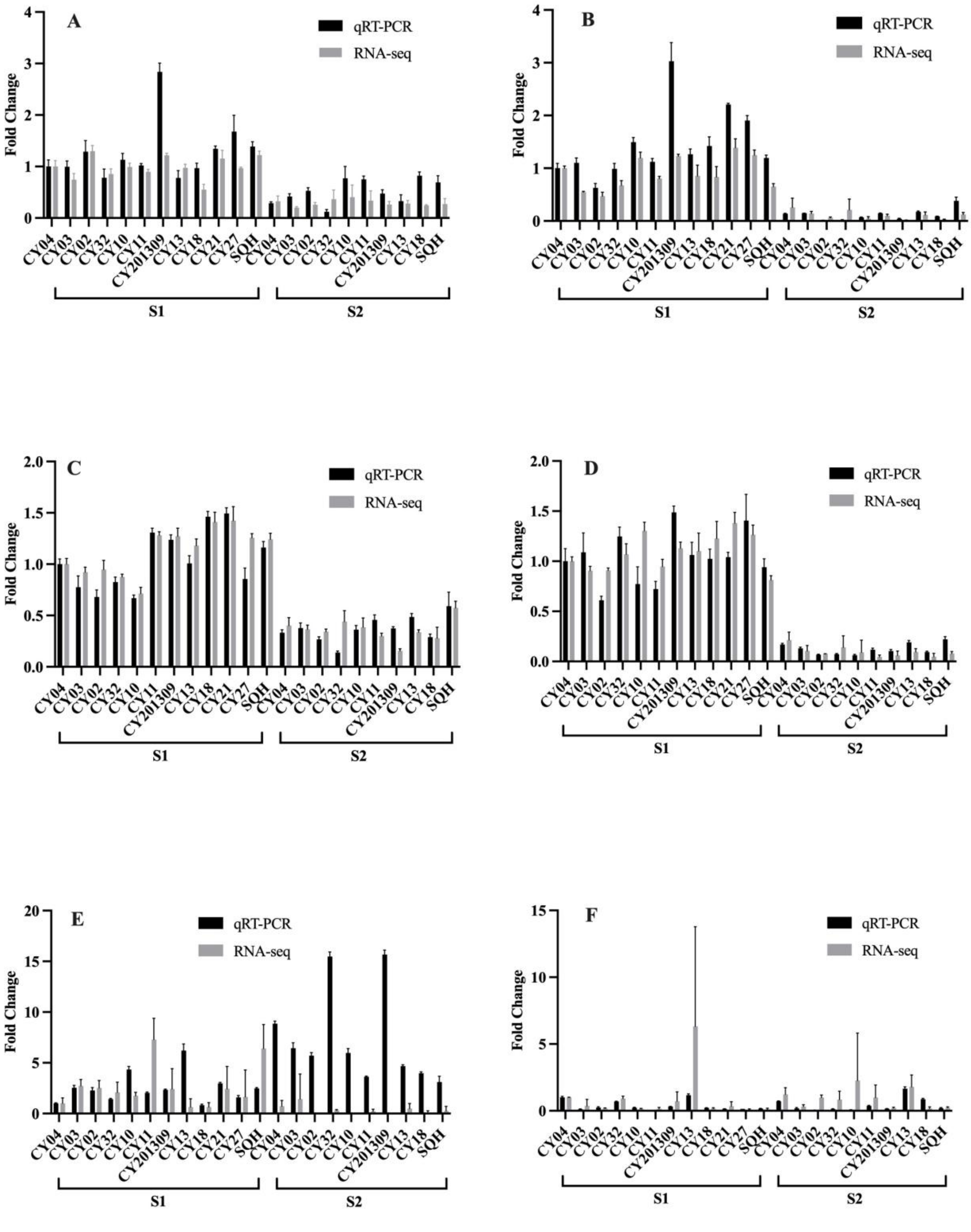

Figure S2. The quantitative real time polymerase chain reaction verification of six hub genes related to lipid biosynthesis. (A) PAP (Hic_asm_12.1324). (B) PAT (Hic_asm_0.74). (C) GPAT6 (Hic_asm_3.866). (D) UPRT (Hic_asm_10.1902). (E) TatD (Hic_asm_0.865). (F) TLP (Hic_asm_18.236).
