# Supplementary material for: Discovery of Hub Genes Involved in Seed Development and Lipid Biosynthesis in Sea Buckthorn (Hippophae rhamnoides L.) Using UID Transcriptome Sequencing
Source: Plants (Basel). 2025 Aug 6;14(15):2436. doi: 10.3390/plants14152436 (PMC12349325; doi:10.3390/plants14152436)
Supplement: Supplementary file 1 [file plants-14-02436-s001.zip › Table S1.pdf]

Table S1 Genes annotation of seed development candidate genes and transcription factors.

Function	Gene name	Gene annotation	Gene id
seed development	FIE	Fertilization independent endosperm	Hic_asm_6.796;Hic_asm_6.797
	HDA	histone deacetylase	Hic_asm_14.1247;Hic_asm_14.1474;Hic_asm_22.807;Hic_asm_6.2774
	SHP	SHATTERPPOOF	Hic_asm_10.530
	CEK	choline/ethanolamine kinase	Hic_asm_22.871;Hic_asm_12.572;Hic_asm_14.114;Hic_asm_20.1236;Hic_asm_20.1237
	MET	methyltransferase 1	Hic_asm_12.2045;Hic_asm_12.2469;Hic_asm_16.1395;Hic_asm_10.1066;Hic_asm_18.2366;Hic_asm_22.1895;Hic_asm_3.1283
	TOR	Target of Rapamycin	Hic_asm_14.284;Hic_asm_6.2621
	UBP	UBIQUITIN-SPECIFIC PROTEASE	Hic_asm_8.401;Hic_asm_10.11;Hic_asm_10.10;Hic_asm_8.1580;Hic_asm_8.1581;Hic_asm_10.1077;Hic_asm_12.1751;Hic_asm_12.2727;Hic_asm_12.2842;Hic_asm_14.1399;Hic_asm_14.1400;Hic_asm_14.156;Hic_asm_14.626;Hic_asm_18.1441;Hic_asm_18.221;Hic_asm_18.386;Hic_asm_18.864;Hic_asm_20.1171;Hic_asm_20.1564;Hic_asm_22.892;Hic_asm_3.577;Hic_asm_5.1443;Hic_asm_5.168;Hic_asm_5.77;Hic_asm_6.1250;Hic_asm_6.1477;Hic_asm_6.3131;Hic_asm_6.99;Hic_asm_8.1495
	DA1	DA1-related protein	Hic_asm_12.858;Hic_asm_16.180;Hic_asm_5.225
	ARF	AUXIN RESPONSE FACTOR	Hic_asm_0.101;Hic_asm_0.988;Hic_asm_10.1391;Hic_asm_10.1827;Hic_asm_12.1206;Hic_asm_12.2861;Hic_asm_14.1054;Hic_asm_14.1145;Hic_asm_14.1146;Hic_asm_16.329;Hic_asm_18.1255;Hic_asm_18.755;Hic_asm_22.206;Hic_asm_22.221;Hic_asm_5.1287;Hic_asm_6.128;Hic_asm_5.1542;Hic_asm_5.1586;Hic_asm_6.2406;Hic_asm_8.1342
	HAIKU	haiku	Hic_asm_0.896;Hic_asm_12.1787;Hic_asm_14.1273;Hic_asm_6.2488;Hic_asm_10.825
	LEC	Leafy Cotyledon	Hic_asm_20.1458
	cesA	cellulose synthase A	Hic_asm_16.1277;Hic_asm_3.15
	TT1	transparent testa 1	Hic_asm_20.739;Hic_asm_8.1048
	NAC	NAC transcription factor	Hic_asm_0.1418;Hic_asm_16.941;Hic_asm_18.1419;Hic_asm_3.686;Hic_asm_5.1348;Hic_asm_6.1385;Hic_asm_8.696;Hic_asm_5.1349
	CKX	cytokinin oxidase/dehydrogenase	Hic_asm_0.1163;Hic_asm_12.2486;Hic_asm_18.2287
	ABA	ABA	Hic_asm_0.1170;Hic_asm_5.100
	BRI1	bri1	Hic_asm_22.1394;Hic_asm_8.524
	SCL	SCARECROW-LIKE	Hic_asm_0.214;Hic_asm_16.1518;Hic_asm_16.715;Hic_asm_18.401;Hic_asm_12.3155;Hic_asm_14.181;Hic_asm_14.25;Hic_asm_20.1443;Hic_asm_3.1602;Hic_asm_0.549
	HPT	histidine phosphotransfer	Hic_asm_12.2202;Hic_asm_3.1833;Hic_asm_3.996;Hic_asm_5.513
	RRS	Response Regulators	Hic_asm_6.3005;Hic_asm_0.758;Hic_asm_12.362;Hic_asm_12.476;Hic_asm_12.49;Hic_asm_14.1395;Hic_asm_16.1470;Hic_asm_18.1065;Hic_asm_20.1213;Hic_asm_22.238;Hic_asm_22.239;Hic_asm_22.661;Hic_asm_3.1243;Hic_asm_3.50;Hic_asm_3.853;Hic_asm_8.1469
	GRAS	GRAS	Hic_asm_22.1159;Hic_asm_22.2078;Hic_asm_6.201
