# Supplementary material for: Discovery of Hub Genes Involved in Seed Development and Lipid Biosynthesis in Sea Buckthorn (Hippophae rhamnoides L.) Using UID Transcriptome Sequencing
Source: Plants (Basel). 2025 Aug 6;14(15):2436. doi: 10.3390/plants14152436 (PMC12349325; doi:10.3390/plants14152436)
Supplement: Supplementary file 1 [file plants-14-02436-s001.zip › Table S2.pdf]

Table S2 Genes annotation of lipid biosynthesis candidate genes and transcription factors.

Function	Gene name	Gene annotation	Gene id
lipid biosynthesis genes	GPD1	Glycerol-3-phosphate dehydrogenase	Hic_asm_12.728;Hic_asm_14.1498;Hic_asm_18.216;Hic_asm_20.1703;Hic_asm_3.490;Hic_asm_22.907;Hic_asm_22.1696
	GPAT	glycerol-3-phosphate 2-O-acyltransferase 6	Hic_asm_12.3049;Hic_asm_22.1358;Hic_asm_22.1105;Hic_asm_22.2250;Hic_asm_3.866;Hic_asm_10.1934;Hic_asm_5.1233;Hic_asm_22.2249;Hic_asm_14.285;Hic_asm_18.1541;Hic_asm_22.402;Hic_asm_12.845;Hic_asm_3.363;Hic_asm_8.866;Hic_asm_6.2722;Hic_asm_8.1089;Hic_asm_5.1345
	LPCAT	lysophosphatidylcholine acyltransferase	Hic_asm_16.399
	LCLAT	lysocardiolipin and lysophospholipid acyltransferase	Hic_asm_8.1438
	DGAT	diacylglycerol acyltransferase 1	Hic_asm_16.1372;Hic_asm_5.1623;Hic_asm_5.287
	PDAT	phospholipid:diacylglycerol acyltransferase	Hic_asm_0.1187;Hic_asm_3.1585;Hic_asm_5.1613
	LCAT	lecithin-cholesterol acyltransferase	Hic_asm_12.1070;Hic_asm_14.862;Hic_asm_18.2240;Hic_asm_3.1362;Hic_asm_6.3037
	BAM	threonine-protein kinase BAM	Hic_asm_0.1956;Hic_asm_5.154
	PGAM	phosphoglycerate mutase	Hic_asm_10.2025
	OLE	oleosin	Hic_asm_18.364;Hic_asm_22.964;Hic_asm_6.1713;Hic_asm_6.2869;Hic_asm_6.844;Hic_asm_8.497;Hic_asm_14.1486
	ACSL	long chain acyl-CoA synthetase	Hic_asm_12.2042;Hic_asm_5.1235;Hic_asm_6.1504;Hic_asm_8.853;Hic_asm_10.1813;Hic_asm_18.1268;Hic_asm_12.2146;Hic_asm_20.2086;Hic_asm_10.633;Hic_asm_22.1181;Hic_asm_0.1016
	ACP	acyl carrier protein	Hic_asm_12.2321;Hic_asm_14.42;Hic_asm_16.624;Hic_asm_3.979;Hic_asm_3.1364;Hic_asm_3.1473
	KCS	ketoacyl-CoA synthase	Hic_asm_0.1261;Hic_asm_0.163;Hic_asm_0.1830;Hic_asm_12.2597;Hic_asm_12.971;Hic_asm_16.1031;Hic_asm_16.1268;Hic_asm_22.1919;Hic_asm_22.252;Hic_asm_3.1164;Hic_asm_5.1091;Hic_asm_5.1720;Hic_asm_8.1177;Hic_asm_8.1545
lipid biosynthesis transcription factors	ABI3	Abscisic acid-insensitive	Hic_asm_8.639;Hic_asm_6.3003;Hic_asm_3.306;Hic_asm_12.257
	AP4	activating enhancer binding protein 4	Hic_asm_10.1874;Hic_asm_0.1139;Hic_asm_6.81;Hic_asm_22.2207;Hic_asm_12.1753
	AP2/ERF	APETALA2/ethylene-responsive element binding factor	Hic_asm_10.1473;Hic_asm_12.2005;Hic_asm_12.2654;Hic_asm_12.2902;Hic_asm_18.172;Hic_asm_20.1674;Hic_asm_22.2165;Hic_asm_3.171;Hic_asm_3.2081;Hic_asm_3.232;Hic_asm_0.1851;Hic_asm_22.643

	FOXO	Forkhead Box Protein O Transcription Factor	Hic_asm_0.25;Hic_asm_10.1543;Hic_asm_12.1171;Hic_asm_12.1353;Hic_asm_12.1354;Hic_asm_12.2359;Hic_asm_12.2449;Hic_asm_16.1553;Hic_asm_16.563;Hic_asm_18.124;Hic_asm_18.2140;Hic_asm_20.1455;Hic_asm_20.1527;Hic_asm_22.140;Hic_asm_3.1119;Hic_asm_3.519;Hic_asm_5.413;Hic_asm_5.972;Hic_asm_6.1719;Hic_asm_6.239;Hic_asm_6.3146
	DAF	DAF Transcription Factor	Hic_asm_18.204
	Dof4	DNA binding with one finger 4	Hic_asm_10.1839;Hic_asm_3.73;Hic_asm_0.851;Hic_asm_12.3128;Hic_asm_0.132;Hic_asm_12.72;Hic_asm_16.672;Hic_asm_18.1733;Hic_asm_18.1888;Hic_asm_20.1563;Hic_asm_20.952;Hic_asm_22.228;Hic_asm_5.589;Hic_asm_5.632;Hic_asm_6.1614;Hic_asm_6.1873;Hic_asm_6.361;Hic_asm_10.1521;Hic_asm_12.1247;Hic_asm_12.1460;Hic_asm_14.243;Hic_asm_16.195;Hic_asm_18.1397;Hic_asm_20.1332;Hic_asm_22.1474;Hic_asm_3.2179;Hic_asm_5.207;Hic_asm_6.66;Hic_asm_8.1454
	Lec1	LEAFY COTYLEDON 1	Hic_asm_0.1591;Hic_asm_3.1713;Hic_asm_3.2231
	WRKY6	WRKY 6 transcription factor	Hic_asm_12.1154;Hic_asm_12.2638;Hic_asm_5.1526
	TCP4	THEOSINTE BRANCH1-CYCLOIDEA-PROLIFERATING CELL FACTOR 4	Hic_asm_22.1558
	WRI1	WRINKLED1	Hic_asm_12.329;Hic_asm_12.626;Hic_asm_18.1664;Hic_asm_3.895;Hic_asm_6.379
	TT2	TESTA 2	Hic_asm_3.1593
	ASIL1	achaete-scute complexlike 1	Hic_asm_12.585;Hic_asm_14.349;Hic_asm_3.632
	SPT5	SPATULA 5	Hic_asm_0.1718;Hic_asm_0.1719;Hic_asm_0.379;Hic_asm_14.215;Hic_asm_18.1194;Hic_asm_18.1449;Hic_asm_22.389
	AGL15	AGAMOUS-LIKE 15	Hic_asm_22.1689
	GRF	Growth-regulating Factor	Hic_asm_16.1067;Hic_asm_22.1377;Hic_asm_16.313
	FUS3	fused in sarcoma	Hic_asm_10.127
	TTG1	TRANSPARENT TESTA GLABRA 1	Hic_asm_3.46;Hic_asm_6.2205
