# Supplementary material for: Discovery of Hub Genes Involved in Seed Development and Lipid Biosynthesis in Sea Buckthorn (Hippophae rhamnoides L.) Using UID Transcriptome Sequencing
Source: Plants (Basel). 2025 Aug 6;14(15):2436. doi: 10.3390/plants14152436 (PMC12349325; doi:10.3390/plants14152436)
Supplement: Supplementary file 1 [file plants-14-02436-s001.zip › Table S3.pdf]

Table S3 Information of all primer sequence in qRT-PCR.

Genes	ID	Forward Primer	Reverse Primer	Size(bp)
PAP	Hic_asm_12.1324	ACCACCACGTCAGCAATCAA	GAGTCGTACGGCTTATCGGG	144
BHLH	Hic_asm_0.1283	GGGCGAAAGAGTTCCCAAGA	TTATTCCCGGGGCTTTGACC	246
PAT	Hic_asm_0.74	CAGGATGTCTCCCGTGCTAC	AGTGACGAACGGTGCAATCT	272
DOF1.2	Hic_asm_12.3128	AGCCCAAAAAGTTCGAGCAC	GCAACTCAAACTCGTGACCC	193
GPAT6	Hic_asm_3.866	TCACAGTCAAAGGAACGCCA	CATCTGTGGCTCTGTCTCGG	216
ERF4	Hic_asm_3.2216	CGAGCTTATGACAACGCTGC	ACCATACACAGCCTCAACCG	285
ACP1	Hic_asm_3.979	GACTCTGCCGTCAATGGTGA	TGCGTCTTGAACCGTGGTAA	147
ATPase	Hic_asm_12.2935	TTGCCGCTATGGGTGTGAAT	CCACGTCTTCCAGGGACTTC	274
UPRT	Hic_asm_10.1902	GGTCGATCCTATGCTTGCGA	GTACCATAGCTGCGGTCTCC	255
TatD	Hic_asm_0.865	TGGGTCGCTAGAGGAGTCAA	AGATGGGCAGAAGTGAAGCC	232
ATPase D	Hic_asm_22.1085	AACTGGATTGGCCAGAGGTG	ACACGACGGTTTGTGGTCTT	144
TLP	Hic_asm_18.2361	CTCGACAACGGCGAAAACTG	CTCTTGTGGACACTGCTCGT	294
